# Do private hospitals outperform public hospitals regarding efficiency, accessibility, and quality of care in the European Union? A literature review

**DOI:** 10.1002/hpm.2502

**Published:** 2018-03-02

**Authors:** Florien M. Kruse, Niek W. Stadhouders, Eddy M. Adang, Stef Groenewoud, Patrick P.T. Jeurissen

**Affiliations:** ^1^ Celsus Academy for Sustainable Healthcare, IQ Healthcare Radboud University Medical Center Nijmegen The Netherlands; ^2^ Department for Health Evidence Radboud University Medical Center Nijmegen The Netherlands; ^3^ Celsus Academy for Sustainable Healthcare, IQ Healthcare, Radboud Institute for Health Sciences Radboud University Medical Center Nijmegen The Netherlands; ^4^ Ministry of Health, Welfare, and Sport The Hague the Netherlands

**Keywords:** efficiency, health care quality, health services accessibility, literature review, private sector

## Abstract

European countries have enhanced the scope of private provision within their health care systems. Privatizing services have been suggested as a means to improve access, quality, and efficiency in health care. This raises questions about the relative performance of private hospitals compared with public hospitals. Most systematic reviews that scrutinize the performance of the private hospitals originate from the United States. A systematic overview for Europe is nonexisting. We fill this gap with a systematic realist review comparing the performance of public hospitals to private hospitals on efficiency, accessibility, and quality of care in the European Union. This review synthesizes evidence from Italy, Germany, the United Kingdom, France, Greece, Austria, Spain, and Portugal. Most evidence suggests that public hospitals are at least as efficient as or are more efficient than private hospitals. Accessibility to broader populations is often a matter of concern in private provision: Patients with higher social‐economic backgrounds hold better access to private hospital provision, especially in private parallel systems such as the United Kingdom and Greece. The existing evidence on quality of care is often too diverse to make a conclusive statement. In conclusion, the growth in private hospital provision seems not related to improvements in performance in Europe. Our evidence further suggests that the private (for‐profit) hospital sector seems to react more strongly to (financial) incentives than other provider types. In such cases, policymakers either should very carefully develop adequate incentive structures or be hesitant to accommodate the growth of the private hospital sector.

## 1 INTRODUCTION

It is an ongoing debate what the role of the private sector in the health care system should be. In theory, under competitive forces and the right preconditions, private hospitals might outperform public providers. However, empirical evidence, mostly originating from the United States, does not confirm such hypothesis.^1^, ^2^, ^3^ For example, Schlesinger and Gray^3^ find that although the evidence is mixed, it seems to favor nonprofit hospitals. Eggleston et al^4^ analyzing differences in quality of care also find mixed evidence. Herrera et al^5^ provide an overview of systematic reviews focusing on quality of for‐profit (FP), not‐for‐profit (NFP), and public providers. Among other things, they concluded that FP providers have higher mortality rates. The US studies illustrate that NFP hospitals seem to mimic FP hospitals on more competitive markets, which might blur the distinctions between both ownership types.^6^

Most European health markets are both less competitive and more inclusive than the United States, which may provide private providers with different incentives. During the past decades, a high amount of public provision spurred discussions about possible inefficiencies, and a movement towards privatization could be observed across Europe.^7^, ^8^ Nowadays, practically all European Union (EU) health systems “contract” both public and private providers. However, EU countries do differ regarding the scale and scope of private hospitals. In most Bismarck‐type systems, private hospitals may be on par with public hospitals: Public and private providers provide comparable services and are reimbursed in a similar way. However, in most Beveridge systems, the private sector runs parallel to the public sector as an alternative provision.^8^ The private sector then also is paid through a parallel private funding scheme (ie, out‐of‐pocket payments or private insurance). Such systematic differences may influence the composition and performance of private hospitals. Furthermore, countries differ on the extent of privatization. In some countries, such as the Nordic countries, hospital ownership is predominantly public, while in other countries, such as the Netherlands, public ownership is nonexistent.

It is currently unknown whether private hospitals outperform public hospitals in the different European health systems. Reviews on this topic are to the best of our knowledge nonexistent. The main aim of this review is to compare the private sector with the public sector on efficiency, quality, and accessibility of services within the EU. We are well aware that the profit status of private hospitals is most likely an important theoretical confounder in explaining differences in performance ever since Arrow^9^ pointed to the fact that private nonprofit status might function as a way to limit market imperfections in situations of unobservable performance of information asymmetries.^9^ However, distinctions between public and private provisions are often at least as important as institutional demarcations, as the distinction between FP and NFP hospitals. That is the reason that we focus on the distinction between public and private. However, if indicated in the included studies, we also differentiate our results between FP and NFP private hospitals.

Our review contributes in 3 ways: (1) to map available literature and to highlight knowledge voids, (2) to identify differences between private and public provisions, and, finally, (3) to find institutional and health care system related drivers for differences in efficiency, accessibility, and quality of care.

## 2 METHODS

### 2.1 Definitions

Public hospitals can be either state owned or fully run by public entities; private ownership can be mission driven (NFP) or return driven (FP).^10^ The term “private” hospitals will be used as an encompassing term throughout this paper, making no distinction between NPF and FP. To compare public and private hospitals, this review will investigate 3 umbrella outcomes: (1) efficiency, (2) accessibility, and (3) quality of care. Efficiency holds the notion as the extent to which objectives are achieved in relation to the resources consumed.^11^ This includes both productivity measures on the basis of frontier analysis or other regression‐based approached, efficiency ratios (eg, employment ratios), and other efficiency outcomes such as length of stay (LOS) or responsiveness to demand. The most applied productivity methods are the stochastic frontier analysis (SFA) and the data envelopment analysis (DEA).^1^, ^12^ Efficiency measures are reflected in multiple indicators such as technical efficiency (maximum output from a given set of inputs or a minimum set of inputs with a given set of outputs), cost efficiency (technical efficiency accounting for the input price), scale efficiency (when the size of the unit is at its optimum), and/or allocative/profit efficiency (cost minimization or profit maximization).^13^ Accessibility is categorized into financial affordability, physical access, informed access, and timely access (eg, waiting times).^14^ Quality of care is structured along the lines of the Donabedian model of structure, process, and outcomes.^15^ Some studied indicators, such as LOS, can be classified under different domains within the Donabedian framework. On the basis of consultations during 2 expert meetings, such indicators were classified towards the most suitable domain. Another difficulty arises with practice variation. To illustrate, does a high rate of surgical interventions indicate better or poor quality of care? To avoid the complex discussion on practice variation and the ambiguous relationship with quality of care, this review does not look into variation in practices.

### 2.2 Realist review

Our study follows a realist review approach. A realistic review is suited to review interventions that are embedded in complex systems, whereby outcomes are dependent and influenced by their contexts.^16^ Rationales and drivers behind the implementation or growth of the private sector are diverse. Because of the peculiar nature of our “intervention,” minor deviations from the realist review protocol were necessary (ie, no explicit distinction is made between intervention, context, and mechanism). This review limits its territory to the EU (28 countries), because the EU countries are, to a certain extent, comparable but have various health care systems. The variety of health care systems can be used to explore how private hospitals perform within various settings. We strive towards a review that “delivers illumination rather than generalizable truths and contextual fine‐tuning rather than standardization.”^16^ ^(p24)^ Hence, the empirical findings are embedded within descriptive context.

### 2.3 Search strategy

The review was conducted from August to October 2015 and updated in June 2017. Data management was done by using Mendeley and Excel. Four databases were searched: Scopus, SocINDEX, Web of Science, and EconLit. Grey literature was excluded. The searches in the relevant databases were updated in June 2017. Different search terms were tested before the actual selection of the articles, to reassure the quality and relevance of the included hits. Table 1 shows the search terms in a simplified manner; in Table A1, the complete search string is given.

**Table 1:** Search terms in abstract, keywords, and title (simplified)

*Intervention:* private hospital OR privatization OR public‐private hospital, OR hospital ownership OR for‐profit hospital
*Outcome:* efficiency OR health care quality OR health care accessibility OR hospital admission OR patient admission OR health care delivery OR affordability OR health care utilization OR health care availability
*AND NOT:* job satisfaction OR Medicare in keywords (for <2008, United States in Keywords)
*Limitations:* Journal articles in English after 2000

### 2.4 Selection process

**Figure 1:**
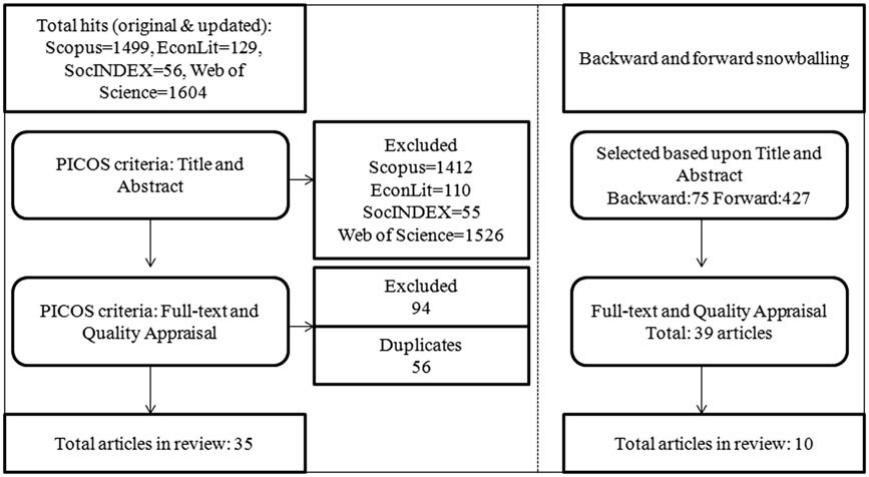
Flow chart of selection process

Figure 1 shows the flow chart of the review process. Only research after 2000, conducted in the EU and articles written in English, were included. Papers were included by matching them with the 5 Population, Intervention, Comparison, Outcome and Study Design (PICOS) criteria (Table 2). To safeguard quality and limit selection bias, the full‐text and appraisal stage was performed by 2 reviewers.

**Table 2:** Inclusion criteria for the second phase

Population	Private hospitals; this could be a nonprofit or for‐profit hospital. Papers that include private hospitals as a control variable are also considered to be eligible.
Intervention/exposure	Patients are exposed to the service delivery of private hospitals.
Comparison	A comparison should be made with public hospitals.
Outcome	One of the following 3 elements should be covered: efficiency, quality of care, and accessibility. Articles that only include employment conditions are not taken into consideration.
Study design	Empirical research, no descriptive papers or economic modeling are included.

Articles were assessed using a standard format to appraise the quality of the studies (see Table A2). The main criteria for exclusion were as follows: (1) research designs were considered to be (extremely) weak and (2) poor reporting on the dataset and methodology, or no possibility of a critical appraisal. The 2 reviewers only included evidence, whereby the quality assessment demonstrated that the findings contributed to our research objective (in Table A3 the excluded references in quality appraisal phase). In total, 35 articles could be included.

A snowballing procedure was performed in December 2015 and January 2016. Forward snowballing identifies articles that refer to the selected articles in the review. Backward snowballing means that the reference list of the articles was included into the review process. Additionally, the literature selected in other systematic reviews covering the EU was included.^1^, ^2^, ^12^, ^17^, ^18^ Such a snowballing methodology has been assessed as a successful addition to the systematic review by advocates of realist reviews.^16^ Articles conceived to be useful upon the PICOS criteria went through the same inclusion process. In total, another 10 articles could be included, bringing the total number of studies to 45.

## 3 RESULTS

The selected articles are shown in summary tables in Table A4. Thirteen articles originated from Italy, 8 from Germany, 7 from the United Kingdom, 6 from France, 5 from Greece, 3 from Austria, 2 from Spain, and 1 from Portugal. While in Germany, Italy, France, and Austria most private hospitals act as a substitute for public hospitals, in the UK, Portugal, Spain, and Greece, most private hospitals do complement the public system.

### 3.1 Efficiency

We found 12 articles using productivity functions assessing primarily technical efficiency. 3 studies analyzing profit and/or cost efficiency, and 10 articles reflecting other efficiency measures (eg, LOS). The evidence on technical efficiency shows no unambiguous conclusion can be made that FP and NFP hospitals are more (cost and/or technical) efficient than public hospitals, although public hospitals seem to be just as efficient as or more efficient than private hospitals. The findings on the other efficiency measures indicate that private hospitals seem to be more responsive to (financial) incentives.

#### 3.1.1 Productivity functions

The studies that estimated technical and/or cost efficiency use a DEA^19^, ^20^, ^21^, ^22^ or an SFA model.^23^, ^24^, ^25^, ^26^, ^27^ Other studies contrast multiple approaches, SFA versus DEA.^28^, ^29^, ^30^ The (adjusted) discharged patients^23^, ^29^ and the number of inpatient (weighted) cases were most often used as output parameters.^20^, ^21^, ^24^, ^25^, ^30^ Diagnosis‐related groups (DRGs),^19^, ^22^ outpatient visits,^19^ and differentiation of specific procedures (eg, number of complex surgery and emergency room treatments)^27^, ^29^ were used less frequently. Regarding input factors, most studies used the number of beds as a proxy for capital investments; one study used the amount spent on supplies as measurement of the capital used.^20^ To identify labor inputs, all studies incorporate the number of full‐time equivalents of physicians, nurses, and other staff members (eg, administrative, nonclinicians, and teaching staff); one study could not include full‐time equivalents, but only the number of staff members because of data limitations.^27^

Only the results on technical efficiency are grouped in Table 3, since this was the dominant outcome and enhances comparability. The findings show mixed results (Table 3), but do indicate more favorable results for public hospitals. Four German studies found that public hospitals were more efficient than FP hospitals.^21^, ^28^, ^30^ One possible explanation is that local governments sell the inefficient hospitals to the private sector.^28^ Also, German FP hospitals with over a thousand beds were found to operate more efficiently.^21^ In Italy, one study found that FP hospitals (Lazio Regio) were less technical efficient than public hospitals.^27^ Whereas when comparing NFP hospitals and public hospitals, the different methodologies and years covered caused divergent results.^27^ Three studies also concluded that NFP hospitals were less efficient in Germany.^21^, ^24^, ^30^ Berta et al^23^ reveal that Italian FP hospitals are less efficient than their public/nonprofit counterparts, but over time have converged towards the same efficiency level as other types. Similar converging results were found in Germany.^25^ NFP hospitals in Germany and Italy also show convergent efficiency scores according to a total of 4 studies.^20^, ^22^, ^23^, ^29^ Two studies, from Austria and Germany, reasoned that private providers are more efficient than public hospitals.^19^, ^20^ The German study analyzed the process of privatization, whereby hospitals that converted to FP status also increased their efficiency. This indicates that a longitudinal design might show different results than cross‐sectional designs. Hospitals that converted to NFP status initially also show increases in efficiency; however, these diminish over time.^20^ In the case of Portugal, one study concludes that private hospitals were more cost‐efficient than their public counterparts.^26^ Using a different methodology—nonoriented super efficiency and different sample selections—no difference in efficiency was found.^22^

**Table 3:** Overview technical efficiency of private hospitals compared with public hospitals

	Less Efficient	No Difference	More Efficient
FP	5 studies from Germany and Italy find private FP hospitals less efficient than public hospitals^21^, ^24^, ^27^, ^28^, ^30^	2 studies from Germany and Italy find no difference between private FP and public hospitals^23^, ^25^	1 study from Germany finds private FP hospitals to be more efficient than public hospitals^20^
NFP	3 studies from Germany find private NFP hospitals to be less efficient than public^21^, ^24^, ^30^	4 studies from Germany and Italy find no difference between private NFP and public hospitals^20^, ^22^, ^23^, ^29^	1 study from Austria finds private NFP hospitals to be more efficient than public hospitals^19^

Abbreviations: FP, for‐profit; NFP, not‐for‐profit.

The overarching message in most studies might actually be the fact that reimbursement schemes are of importance. In Italy, FP hospitals were found to be less efficient because they use resources less efficiently. This might be due to the fact that private FP hospitals are confronted with specific regulations that set a limit to the number of funded admissions; since such limits fluctuate over time and are quite volatile, FP hospitals might face problems to adjust fixed input resources accordingly.^27^ Another indication of the importance of funding schemes might be the fact that after a DRG‐based payment system had been introduced in Italy, NFP hospitals converged to the same levels of technical efficiency as public hospitals.^29^ In Germany, Herr et al^25^ also found no statistically significant differences in technical efficiency between FP and public hospitals after a DRG‐based payment system had been introduced in 2004. Earlier, Herr^24^ showed that private hospitals were on average less cost and technical efficient, maybe because of the fact that in that timeframe, there existed an incentive to increase LOS to raise revenues. Nonetheless, FP hospitals were found to be more profit efficient than public hospitals, meaning that hospitals have certain output prices and input prices, and FP hospitals choose the best combination of both input and output factors.^25^ However, another study discovered that under the DRG payment system, efficiency gains among FP‐privatized hospitals were significantly lower compared with before the DRG payment system.^20^ The Austrian DRG system only covers up to 50% of hospital costs, and additional funds come from states and operational‐deficit coverage, determined ex post by the local authorities. Such funds disproportionally accrue to public providers placing the private sector at bay, but possibly also increasing their incentives to operate more cost conscious.^19^

#### 3.1.2 Other efficiency outcomes

A subset of studies do use other outcomes to assess the efficiency of hospital providers. Multiple studies analyze the relationship between ownership and LOS (Table 4). A short case‐mixed LOS is seen as an indicator of superior efficiency. French private hospitals have longer LOS for knee procedures, but shorter LOS for hip procedures.^31^ For most diagnostic groups, there exists no difference in LOS between UK public hospitals and private independent sector treatment centers (ISTCs), although for some treatments, particularly hip and knee procedures, a longer LOS was found for National Health Service (NHS) hospitals.^32^ Another study using the same dataset as the former study supports the latter findings, whereby LOS in ISTCs is shorter than in public hospitals for hip replacements.^33^ Evidence from Italy reports shorter LOS in private hospitals for aortic valve substitution.^34^ However, LOS was found to be longer in Italian private psychiatric hospitals.^35^ The authors explain this by private psychiatric hospitals being funded on a per diem basis, creating incentives to increase LOS. Indeed, in Greece, LOS was also higher in private mental health clinics.^36^ This alludes to the assumption that FP providers seem to apply more revenue‐maximizing strategies. Overall, per diem funding structures—as in mental health—seem to increase LOS among private providers, while prospective structures as in acute care seem to create an opposing effect. Both underline the idea that the private providers respond more intensely to incentives than public hospitals. This is tested in a more head‐to‐head approach by Schwierz.^37^ The author identifies that the introduction of a new payment system in 2014 pushed for economic discipline and penalized high‐cost hospitals, creating incentives for German private hospitals to take over public hospitals.^37^ In general, FP hospitals were also found to respond faster to increasing demand than other ownership types. Public hospitals were more likely to default; therefore, privatization became an appealing option.^37^ Another study, also conducted in Germany, analyzes changes in hospital staff after privatization. This study discovers that FP privatization reduced staff per inpatient case (especially nurses, other nonphysician clinical staff, and other nonclinical staff). Such findings were not found when NFP hospitals were the acquiring party.^38^ Similar finding was found in Greece; FP hospitals seem to have lower nursing staff rates for nurses compared with the public hospitals.^36^

**Table 4:** Other efficiency measures

Outcome/Indicator	Number of Studies	Type (Private)	Countries	Impact
LOS	3	Aortic valve substitution, hip and knee procedures in private hospitals or ISTCs	Italy, United Kingdom, France	Private hospitals have shorter LOS
	3	Private (ie, psychiatric hospitals, mental health clinics) hospitals and specifically for knee procedures	Italy, Greece, France	Private hospitals have longer LOS
	1	ISTCs (for most diagnostic groups)	United Kingdom	No difference
Responsiveness to demand	1	FP	Germany	Public hospitals are less responsive
Employment	1	NFP	Germany	No difference
	2	FP	Germany, Greece	Lower staff rate
Upcoding	1	NFP + FP	Italy	Public hospitals have less “upcoding”
	1	NFP + FP	Italy	No difference

Abbreviations: ISTCs, independent sector treatment centers; FP, for‐profit; LOS, length of stay; NFP, not‐for‐profit.

Finally, 2 studies addressed upcoding. In Italy, Vittadini et al^39^ looked at registering patients with nonexisting complications to increase reimbursement. There was evidence that both NFP and FP hospitals were to some extent engaged in “upcoding” before a specific law against upcoding in 2007 was institutionalized. No such evidence was found for public hospitals.^39^ Berta et al^23^ also found that during 2003 to 2005, FP hospitals had more intense upcoding practices than other hospital types. However, no ownership differences were found after 2005, probably because of more severe checks implemented after 2003.^23^

### 3.2 Accessibility

Included articles examine 11 different indicators of accessibility (Table 5). Most included studies do raise concerns about accessibility to private hospitals; most of them flag this issue by analyzing the complexity of the cases and various patients' characteristics. In many countries, private providers do target higher socioeconomic classes, often through parallel private insurance. High‐income patients hold better access to private hospitals and that waiting times in the private sector are lower.

**Table 5:** Accessibility indicators overview

Concept	Number of Studies	Outcome/Indicator	Type (Private)	Countries	Impact
Affordable	8	SES of patients (eg, employment status, residents from deprived versus affluent region)	Private (ie, maternity, psychiatric), ISTCs	Italy, United Kingdom, Greece, Spain	Public hospitals perform better
	2	Method of payment (ie, private health insurance and pay out‐of‐pocket)	Private	Greece	
	1	Payment per discharge	FP	Greece	
Physical	3	Case‐mix differences (eg, cream skimming)	FP, ISTCs	Italy, UK	
	1	Access to specialty care (ie, adjusted rates of revascularization)	Private	France	
	1	Admission pattern	Private psychiatric	Italy	
	1	Access to preemptive registration	FP	France	
	1	Regional physical mobility (number of nonresident patients in the region admitted)	Private	Italy	

Physical	1	Mean expenditure and usage of drugs	FP	France	No difference
Affordable	1	Access to specialty care (ie, ambulatory care services)	Private	France	Private hospitals perform better
	1	Method of payment (ie, informal payment)	Private	Greece	
Physical	1	Chance op follow‐up treatment	Private psychiatric	Italy	
Timely	1	Waiting times	ISTCs	UK	

Abbreviations: ISTCs, independent sector treatment centers; FP, for‐profit; SES, socioeconomic status.

#### 3.2.1 Affordable access

In the United Kingdom, patients of private ISTCs are less likely to coming from deprived residential areas.^32^, ^40^ One other study concludes that patients in private hospitals diagnosed with prostate cancer come from the more affluent regions.^41^ In Greece, monthly family income is positively related to private hospital admissions.^42^, ^43^, ^44^ In addition, both patients with private health insurance and rural residents are more likely to use private care services.^44^ Under comparable circumstances, FP hospitals generally charge more for admitted patients falling under the Greek Social Health Insurance fund.^36^ In Greece, more private patients had to pay out‐of‐pocket payments than in public hospitals. On the other hand, and maybe remarkably, “under‐the‐table” payments were lower in private hospitals.^45^

In Spain, private maternity units/hospitals proportionally treat more patients from higher socioeconomic backgrounds.^46^, ^47^ In private hospitals, the prevalence of cesarean sections was also higher among immigrants in comparison with natives; no such distinctions were found within public hospitals.^47^ In Italy, patient characteristics differ between private and public (psychiatric) hospitals. Older patients are less likely to be unemployed and make more use of private services.^48^

#### 3.2.2 Physical access

Private hospitals are often accused of cream skimming and selecting more profitable patients. We found some illustrations to that suspicion. One Italian study argues that FP hospitals were more involved in cream skimming than both public or NFP hospitals.^23^ In the United Kingdom, ISTCs treat less complex NHS patients.^32^, ^40^ In France, a higher percentage of patients with ambulatory care sensitive conditions visit public hospitals in comparison with private hospitals, while the opposite appears for revascularization. The explanation is that in France, public and NFP hospitals account for most acute inpatient stays and FP hospitals provide half the total revascularizations procedures.^49^ Regarding a specific case from Italy, Preti et al^50^ detected that private psychiatric facilities were less likely to admit patients who attempted suicide prior to admission; this might serve as an indicator that high‐risk mental health patients are less able to access private services. Patients in private acute psychiatric inpatient clinics were also more likely to receive a follow‐up treatment (ie, rehabilitation and psychotherapy).^48^ Bonastre et al^51^ identified that in France, no significant differences exist between public and private hospitals in relation to the use of expensive drugs (anticancer drugs), after controlling for case mix. One French study investigated if hospital types differed in terms of access to renal (kidney) transplantation. The authors observe that FP hospitals were less likely to have patients on the preemptive registration list than (public) academic hospitals, corrected for case‐mix differences.^52^ Preemptive transplantation is associated with longer patient survival. Hence, patients in FP hospitals might be disadvantaged in access to such treatments. Regarding regional mobility, a study from Italy found that nonresident patients are more likely to be admitted to private hospitals compared with public hospitals when they could not gain access to care in their own region.^34^ The authors point out that this is of concern, since patients with financial resources can afford to be more mobile.^34^

#### 3.2.3 Timely access

In the United Kingdom, shorter inpatient waiting times are associated with higher rates of private hospital beds.^53^

### 3.3 Quality of care

Quality of care encompasses many different aspects of health care. This is also reflected in the variety of outcome variables found in this review (Table 6). The quality of care studies are structured according to the Donabedian model of structure, process, and outcomes^15^ and show mixed results.

**Table 6:** Quality of care indicators overview

Concept	Number of Studies	Outcome/Indicator	Type (Private)	Country	Impact
Structure	1	Discontinuity of care	Private psychiatric	Italy	Public hospitals perform better
	1	Qualification staff	FP	Greece
Process	2	Adherence guideline and screening	Private	Austria and Italy	
	1	Appropriate admission	Private	Italy	
Outcome	2	Mortality rate (avoidable mortality)	FP, private	France, Italy	
	1	Rehospitalization rates	Private	France	

Outcome	1	Patient's experiences	ISTCs	United Kingdom	No difference
Outcome	3	Mortality (risk of dying)	Private hospitals, NFP and FP	Germany, Italy	Private hospitals perform better
	1	Readmission (likely to be readmitted in 30 days)	Private hospitals	Italy	
	1	Patients experience (regarding amenities)	ISTCs	United Kingdom	

Abbreviations: ISTCs, independent sector treatment centers; FP, for‐profit; NFP, not‐for‐profit.

#### 3.3.1 Structure

Kondilis et al^36^ find that FP hospitals in Greece seem to have less qualified compared with the public hospitals. One of the possible explanations given by the authors is that FP hospitals might maximize profits and therefore minimize expenses on nursing staff. Another possible explanation is that FP hospitals use nursing staff more efficiently than public facilities. In Italy, private psychiatric clinics collaborated less intensely with the community system as public psychiatric departments do.^48^

#### 3.3.2 Process

From discharge data extracted from Emilia‐Romagna hospitals, the appropriateness of admission was evaluated. Although the number of inappropriate admissions decreased between 2001 and 2005, private hospitals exhibit in all years more inappropriate admissions than public hospitals.^54^ Private hospitals are also showing less adherence to antenatal screening among pregnant women in 6 Italian regions.^55^ A study on Austrian hospitals shows that adherence to the guidelines for colorectal cancer screening was worse among private hospitals. After the implementation of a guideline for colorectal screening, only 3.8% of private hospitals changed their routine practice versus 14.2% of public hospitals.^56^

#### 3.3.3 Outcomes

In Germany, Tiemann and Schreyögg^21^ analyzed hospital mortality rates. They found that, controlling for case‐mix differences, FP and NFP hospitals showed better mortality figures than the public sector. One of the potential explanations for this finding might be that publicly enforced transparency on quality indicators seems to have stimulated FP hospitals to put comparatively more emphasis on such issues.

France was the country were the 2 included studies on quality outcomes indicated a consistently worse performance for the private sector. Mortality rates for patients aged over 35 and admitted for heart attacks were found to differ among hospital types. Public (nonteaching) hospitals have a lower mortality rates compared with FP hospitals.^57^ Rehospitalization rates, a possible indicator for worse quality, differ as well between French hospitals. Private hospitals have higher rates of 30‐day all‐cause rehospitalizations of older patients compared with public providers.^58^

In Italy, regional degrees of privatization (1993‐2003) are used as a quasinatural experimental design to investigate the association between public and private hospitals spending on (the reduction of) avoidable mortality. Spending increases on public delivery of health care services was associated with increased reduction in avoidable mortality. However, no such positive effects were found with respect to spending increases on private health care services. This implies that increases of spending on private health care services might hamper the possible reduction in avoidable mortality by investments in the public sector.^59^ Contrary results indicate that patients in private hospitals are less likely to be readmitted and less likely to die within 30 days after discharge, although the impact of the latter was found to be much lower.^60^ This corresponds to the results of a multilevel analysis, also from Italy, which assessed that the risk of dying was significantly less in private hospitals.^61^

Both Pérotin et al^62^ and Owusu‐Frimpong et al^63^ examine UK patient experiences. The latter study finds that users of ISTCs have higher satisfaction rates than the users of public facilities for amenities, for instance, obtaining attention from doctors.^63^ However, Pérotin did not find a significant difference on the reported overall patient experiences between public and private clinics. Differences that were found seemed to relate to other variables such as patient characteristics.^62^

## 4 DISCUSSION AND CONCLUSION

This review points to various messages. Findings on efficiency show mixed results, but do suggest that the public sector is at least as or more efficient as the private sector. Many papers mention that the institutional context might be an important constraint for the efficiency for the private sector. For example, Austrian NFP hospitals seem to be “induced” to operate with high levels of operational efficiency. There exists quite some evidence that the private sector seems more sensitive to incentives than the public sector. This was shown for a range of indicators such as responding to changes in demand, upcoding, or adjusting LOS. Differences in LOS seem to depend on type of treatment, whereby consistent evidence shows the private sector has shorter LOS for hip procedures compared with the public sector and type of payment: Per diem funding increases LOS in private settings more than in public surroundings, especially for mental health. As expected, in South European countries and also in the United Kingdom where a parallel and partly duplicate system exists between private and public provisions, the private sector is used by the more affluent population, who may experience, for example, lower waiting times and better amenities. This suggests that universal access and a broader inclusion of private providers in the mainstream health system might be an important option to reduce such disparities in access. The same goes for cream‐skimming, which, although higher in private hospitals, might be prevented by sophisticated case‐mix corrections in the payment structures. Private hospitals may perform better on observable quality outcomes such as for example exist in Germany and Italy for mortality and readmissions. In France, private hospitals specialize in certain (elective) procedures. One might expect better outcomes for private hospitals as a result of such specializations, but in France, the findings predominantly seem to favor public hospitals. This casts doubt on the advantages of private hospital specialization.

This realist review analyzes a complex and context‐dependent issue and thus is subject to various limitations. Included studies used a wide range of indicators; research designs vary substantially. This makes it somewhat problematic to extrapolate or generalize these findings. Many findings relate to specific diseases and/or indicators implying they do not necessarily hold for a broader spectrum of diseases. Studies covering efficiency showed more consistency among their use of parameters and methodology. We also were able to only include studies from a limited number of EU countries. Most evidence compromises a few countries: Italy, Germany, France, the United Kingdom, and Greece. However, these 5 countries do cover for a substantial part of the total EU population and—more importantly—cover for most health care system types (tax‐funded or social insurance, multiple payer and single payer, and decentralized and more centralized). Including articles not written in English could broaden the scope of this research. Furthermore, transferability of our results from one country to another is a difficult and complex task.^64^ The performance of different types of hospital ownership may be highly dependent on their embeddedness in health system ecosystems. Indeed, private hospitals may compete, specialize, or complement public providers, which could partly explain conflicting outcomes. A more thorough understanding of the position of the private sector in the wider health system could aid policy makers in designing sound and evidence‐based policies in this area.

We provide policymakers with several take‐away messages. Firstly, the private hospital sector consists of many complex layers. Both a polarizing political debate and traditional economist reasoning towards the superiority of a regulated market also in health care do not suit the complexity of the issue. Secondly, our evidence shows that one should take a careful note to the incentives built into the health care systems, because they seem to be an important driver for either the divergence or convergence of the private and public sector. For‐profit providers seem to respond more intensely to incentives. Fine tuning such structure, eg, hospital payment systems, becomes even more important if the role of the private sector increases. Thirdly, despite popular opinion that enhancing the role of the private sector increases efficiency, we do not find a lot of evidence that supports this claim. Most evidence shows that public hospitals are as efficient as or more efficient than private counterparts. For Beveridge countries, we found that access to private hospitals is substantially worse for patients with either low incomes or a more complex case mix. Finally, this review highlights that policy “shopping” among research results is dangerous. The evidence on private sector performance should be critically assessed; research designs (ie, indicator specification, methodology, and sample selection) do cause divergent results between studies. Our assessment is that the supposed superior performance of the private sector—and especially the private nonprofit hospital sector—for Beveridge countries depends on full inclusion in the health system to guarantee broader access to the private sector.

Overall, this review could contribute to the discussion on the role of the private sector in providing hospital services in the EU and how different systems, institutions, and incentive structures might affect the public and private hospital sectors.

## ETHICS STATEMENT

No ethical approval was required for this research, since this research is based on review of published literature.

## APPENDIX A

### A.1

**Table A1:** Search string

Scopus	
Before 2008	
**Search in title, abstract, and key** Block 1: (private ‐within 2 words‐ hospital) AND efficiency OR “health care quality” OR “quality of health care” OR (health care ‐within 3 words‐ access*) OR “hospital admission” OR “patient admission” OR afford* OR “health care ‐within 3 words‐ delivery” OR “health care utilization” OR “health care availability” AND NOT “job satisfaction” OR Block 2: hospital AND privatization AND efficiency OR “health care quality” OR “quality of health care” OR (health care ‐within 3 words‐ access*) OR “hospital admission” OR “patient admission” OR afford* OR “health care ‐within 3 words‐ delivery” OR “health care utilization” OR “health care availability” AND NOT “job satisfaction” OR Block 3: (“public private*” ‐within 3 words‐ hospital) AND efficiency OR “health care quality” OR “quality of health care” OR (health care ‐within 3 words‐ access*) OR “hospital admission” OR “patient admission” OR afford* OR “health care ‐within 3 words‐ delivery” OR “health care utilization” OR “health care availability” AND NOT “job satisfaction” OR Block 4 “hospital ownership” AND efficiency OR “health care quality” OR “quality of health care” OR (health care ‐within 3 words‐ access*) OR “hospital admission” OR “patient admission” OR afford* OR “health care ‐within 3 words‐ delivery” OR “health care utilization” OR “health care availability” AND NOT “job satisfaction” Block 5 “for profit hospital” AND efficiency OR “health care quality” OR “quality of health care” OR (health care ‐within 3 words‐ access*) OR “hospital admission” OR “patient admission” OR afford* OR “health care ‐within 3 words‐ delivery” OR “health care utilization” OR “health care availability” AND NOT “job satisfaction” And no keywords “Medicare” OR “US” OR “United States” Limit to Journal, Article, English	
After 2008	
**Search in title, abstract, and key** Block 1: (private ‐within 2 words‐ hospital) AND efficiency OR “health care quality” OR “quality of health care” OR (health care ‐within 3 words‐ access*) OR “hospital admission” OR “patient admission” OR afford* OR “health care ‐within 3 words‐ delivery” OR “health care utilization” OR “health care availability” AND NOT “job satisfaction” OR Block 2: hospital AND privatization AND efficiency OR “health care quality” OR “quality of health care” OR (health care ‐within 3 words‐ access*) OR “hospital admission” OR “patient admission” OR afford* OR “health care ‐within 3 words‐ delivery” OR “health care utilization” OR “health care availability” AND NOT “job satisfaction” OR Block 3: “public private*” ‐within 3 words‐ hospital) AND efficiency OR “health care quality” OR “quality of health care” OR (health care ‐within 3 words‐ access*) OR “hospital admission” OR “patient admission” OR afford* OR “health care ‐within 3 words‐ delivery” OR “health care utilization” OR “health care availability” AND NOT “job satisfaction” OR Block 4 “hospital ownership” AND efficiency OR “health care quality” OR “quality of health care” OR (health care ‐within 3 words access*) OR “hospital admission” OR “patient admission” OR afford* OR “health care ‐within 3 words‐ delivery” OR “health care utilization” OR “health care availability” AND NOT “job satisfaction” Block 5 “for profit hospital” AND efficiency OR “health care quality” OR “quality of health care” OR (health care ‐within 3 words‐ access*) OR “hospital admission” OR “patient admission” OR afford* OR “health care ‐within 3 words‐ delivery” OR “health care utilization” OR “health care availability” AND NOT “job satisfaction”	
And no keywords “Medicare”	
Limit to Journal, Article, English	
Search string: EconLit & SocINDEX	
Search terms (AB “private w/2 hospital” OR AB (privatization AND hospital**)** OR AB “hospital ownership” OR AB “for profit hospitals” OR AB “public private w/3 hospital” OR AB “PPP w/3 hospital”) OR (SU (“private w/2 hospital” OR (privatization AND hospital) OR “hospital ownership” OR “for profit hospitals” OR “public private w/3 hospital” OR AB “PPP w/3 hospital”)	Search Options Published Date: 20000101‐20151231 Source types Academic Journals and English
Search string: Web of Science	
TS = “private hospital” OR TS = (privatization AND hospital) OR TS = “hospital ownership” OR TS = “for profit hospital” OR TS = “non profit hospital” OR TS = (“public private” AND hospital) OR TS = (PPP AND hospital) AND LANGUAGE: (English) AND DOCUMENT TYPES: (Article)	Indexes = SCI‐EXPANDED, SSCI, A&HCI, ESCI Timespan = 2000‐2017

**Table A2:** Quality appraisal form

Component Ratings of Study:	Score	Justification/Comments
Strong = 3/Modest = 2/Weak = 1		
**A) Design**		
Outcome of interest as main (3) or control variable (2/1)?		
Cross‐sectional (2/1) or longitudinal (3)		
Prospective (3) or retrospective (2/1)		
Is the method of analysis appropriate? (strong, modest, weak)		
Is the method of analysis sufficiently rigorous? (strong, modest, weak)		
**B) Quality of reporting**		
Enough data have been presented to show how the authors arrived at their findings (Strong, Modest, Weak)		
Enough information is given what the methodological design is? (Strong, Modest, Weak)		
Enough information is given where the data comes from and what the characteristics are of the sample (ie, summary statistics and sample sizes). (Strong, Modest, Weak)		
**C) Selection bias**		
Strong: The selected individuals/hospitals are very likely to be representative of the target population		
Moderate: The selected individuals/hospitals are at least somewhat likely to be representative of the target population		
Weak: The selected individuals/hospitals are not likely to be representative of the target population		
**D) Confounders (ie, region, demographics)**		
Strong: will be assigned to those articles that controlled for most relevant confounders		
Moderate: will be given to those studies that controlled for relevant confounders, but explicitly mentions that it missed some relevant confounders		
Weak: will be assigned when the relevant confounders were not controlled for		
**E) Data collection methods**		
Strong: The data collection tools have been shown to be valid; and the data collection tools have been shown to be reliable		
Moderate: The data collection tools have been shown to be valid; and the data collection tools have not been shown to be reliable or reliability is not described.		
Weak: The data collection tools have not been shown to be valid or both reliability and validity are not described.		
**F) Outcome variable**		
*The choice of measurement of the outcome variable (accessibility, quality of care efficiency) is valid?*		
Strong: Clear connection with 1 of the 3 concepts, and/or is generally accepted by scholars		
Moderate: A couple of validity issues arise. The connection between the outcome variable and the concepts of interest is moderate (eg, only one disease is analyzed)		
Weak: Serious concerns about how the outcome variable (1 of the 3 concepts) is measured		
**G) Number of hospitals**		
Strong: More than 10 hospitals are included in the analysis		
Moderate: Between 3 and 10 hospitals are included in the analysis		
Weak: Only 2 hospitals are compared		
**H) Context**		
Strong: Includes many different contexts/regions, high complexity in demographic characteristics		
Moderate: Combines 2 or 3 different regions		
Weak: One very specific region with specific characteristics		
**J) Independence**		
Is this an independent study? Yes (3) Debatable (2) No (1)		
**K) Drop‐outs—only if applicable**		
Strong: (If applicable: will be assigned when the follow‐up rate is 80% or greater).		
Moderate (If applicable: will be assigned when the follow‐up rate is 60%‐79%).		
Weak: (If applicable: will be assigned when a follow‐up rate is less than 60% or if the withdrawals and drop‐outs were not described).		
	Total score	
**Additional comments**		**Answers to comments**
Do the results seem to be valid?		
Do the results seem to be reliable?		
Are the results relevant? Does it fall within the scope of our research question?		
Can the results be generalized?		
	In or out	If needed: justification
Final judgment made based on the score and the additional comments		

**Table A3:** Excluded references in quality appraisal

Browne, J., L. Jamieson, J. Lewsey, J. van der Meulen, L. Copley and N. Black (2008). “Case‐mix & patients' reports of outcome in Independent Sector Treatment Centres: comparison with NHS providers.” BMC health services research **8**: 78. Caballer‐Tarazona, M., A. Clemente‐Collado and D. Vivas‐Consuelo (2016). “A cost and performance comparison of public private partnership and public hospitals in Spain.” Health Economics Review **6**(1): 1‐7. Colais, P., L. Pinnarelli, D. Fusco, M. Davoli, M. Braga and C. A. Perucci (2013). “The impact of a pay‐for‐performance system on timing to hip fracture surgery: experience from the Lazio Region (Italy).” BMC health services research **13**: 393. De Girolamo, G., A. Barbato, R. Bracco, A. Gaddini, R. Miglio, P. Morosini, B. Norcio, A. Picardi, E. Rossi and P. Rucci (2007). “Characteristics and activities of acute psychiatric in‐patient facilities: national survey in Italy.” The British Journal of Psychiatry **191**(2): 170‐177. Grilli, R., P. Guastaroba and F. Taroni (2007). “Effect of hospital ownership status and payment structure on the adoption and use of drug‐eluting stents for percutaneous coronary interventions.” Canadian Medical Association Journal **176**(2): 185‐190. Keong, N., D. Ricketts, N. Alakeson and P. Rust (2004). “Pressure sores following elective total hip arthroplasty: pitfalls of misinterpretation.” Annals of the Royal College of Surgeons of England **86**: 174‐176. Kontodimopoulos, N., P. Nanos and D. Niakas (2006). “Balancing efficiency of health services and equity of access in remote areas in Greece.” Health policy (Amsterdam, Netherlands) **76**: 49‐57. Mathoulin‐Pélissier, S., Y. Bécouarn, G. Belleannée, E. Pinon, A. Jaffré, G. Coureau, D. Auby, J.‐L. Renaud‐Salis and E. Rullier (2012). “Quality indicators for colorectal cancer surgery and care according to patient‐, tumor‐, and hospital‐related factors.” BMC cancer **12**: 297. Mossialos, E., S. Allin, K. Karras and K. Davaki (2005). “An investigation of caesarean sections in three Greek hospitals: the impact of financial incentives and convenience.” European journal of public health **15**: 288‐295. Nemec, J., B. Meričková and J. Štrangfeldová (2010). “The ownership form of hospitals from the viewpoints of economic theory and slovak practice.” E a M: Ekonomie a Management **13**: 19‐31. O' Herlihy, A., P. Lelliott, D. Bannister, A. Cotgrove, H. Farr and S. Tulloch (2007). Provision of child and adolescent mental health in‐patient services in England between 1999 and 2006. Psychiatric Bulletin. Papadaki, S. and P. Stankova (2016). “Comparison of horizontally integrated hospitals in private and public sectors of Czech Republic.” Economics and Sociology **9**(3): 180‐194. Sellbrant, I., C. Pedroletti and J. G. Jakobsson (2017). “Pelvic organ prolapse surgery: changes in perioperative management improving hospital pathway.” Minerva Ginecologica **69**(1): 18‐22. van Rompaey, B., M. M. Elseviers, M. J. Schuurmans, L. M. Shortridge‐Baggett, S. Truijen and L. Bossaert (2009). “Risk factors for delirium in intensive care patients: a prospective cohort study.” Critical Care **13**(3). Vanhegan, I., A. Hakmi, N. De Roeck and A. Rumian (2015). “Effect of an independent‐sector treatment centre on provision of elective orthopaedic surgery in East and North Hertfordshire.” Annals of the Royal College of Surgeons of England **97**(7): 519‐525. Wood, G. C. a. and C. Howie (2011). “Do waiting list initiatives discriminate in favour of those in a higher socioeconomic group?” Scottish Medical Journal **56**: 76‐79.

**Table A4:** Summary of findings table (alphabetic order)

	Indicator	Methodology	Reliability Results	Generalizability Results	Year(s) Covered	Type (Private)	Country
**Efficiency**							
Barbetta et al^29^	Technical	COLS, SFA, and DEA	Strong	Strong	1995‐2000	NFP	Italy
Barros et al^26^	Cost	SFA	Moderate	Moderate	1997‐2008	NS	Portugal
Czypionka et al^19^	Technical	Two‐stage DEA	Strong	Strong	2010	NFP	Austria
Daidone and D'Amico^27^	Technical	SFA	Strong	Moderate	2000‐2005	FP + NFP	Italy
Gigantesco et al^35^	LOS	Logistic regression	Weak	Strong	2002‐2003	Psych.	Italy
Tiemann and Schreyogg^20^	Technical	Two‐stage DEA and Diff‐in‐Diff	Strong	Strong	1996‐2008	FP + NFP	Germany
Heimeshoff, Schreyögg, and Tiemann^38^	Employment reduction	Diff‐in‐Diff and FE	Strong	Strong	1996‐2008	FP + NFP	Germany
Herr^24^	Technical and cost	SFA	Strong	Strong	2000‐2003	FP + NFP	Germany
Herr, Schmitz, and Augurzky^25^	Technical, cost and profit	SFA	Strong	Strong	2002‐2006	FP	Germany
Herwartz and Strumann^28^	Technical	Two‐stage DEA + SFA	Strong	Strong	1995‐2008	FP + NFP	Germany
Lindlbauer and Schreyögg^30^	Technical	Two‐stage DEA and SFA	Strong	Strong	2000‐2010	FP + NFP	Germany
Maravic and Landais^31^	LOS	Linear multiple regression	Weak	Weak	2001	NS	France
Schwierz^37^	Responsiveness to demand changes	IVs + FE	Strong	Strong	1996‐2006	FP + NFP	Germany
Siciliani et al^33^	LOS	Quantile regression	Moderate	Weak	2006‐2007	NS	United Kingdom
Sommersguter‐Reichmann and Stepan^22^	Technical	Super efficiency DEA	Strong	Strong	2009‐2012	NFP	Austria
Vittadini et al^39^	Upcoding (by the LOS)	Diff‐in‐Diff and FE	Strong	Moderate	2007‐2008	FP + NFP	Italy
**Accessibility**							
Barbiere et al^41^	Utilization by socioeconomic status	Multivariate logistic regression	Moderate	Weak	1998‐2006	ISTC	United Kingdom
Biro and Hellowell^53^	Waiting times	Region fixed effects	Moderate	Strong	2000‐2001 & 2008‐2009	ISTC	United Kingdom
Bonastre et al^51^	Mean expenditure and usage chemotherapy	Multilevel analysis	Strong	Strong	2008	FP	France
Gusmano et al^58^	Avoidable hospitalization	Multilevel analysis	Strong	Strong	2004‐2008	NS	France
Mason et al^40^	Patient complexity	Mean difference by HRG	Moderate	Low	2005‐2006 & 2006‐2007	ISTC	United Kingdom
Pappa et al^42^	Utilization by socio economic status	Multivariate logistic	Moderate	Low	2003	NS	Greece
Preti et al^50^	Admission after suicide	Multivariate logistic	Moderate	Low	2004	Psych.	Italy
Riffaut et al^52^	Access to preemptive registration on the waiting list	Multilevel analysis	Strong	Low	2008‐2012	FP	France
Río et al^46^	Utilization by socioeconomic status	Logistic regression	Low	Moderate	2005‐2006	NS	Spain
Salvador et al (2009)	Utilization by socioeconomic status	Logistic regression	Low	Low	1993‐2003	NS	Spain
Siskou et al^43^	Utilization by socioeconomic status and rural versus urban citizens	Stratified survey‐logistic regression	Low	Moderate	2005	NS	Greece
Souliotis et al^45^	Utilization by socioeconomic and out‐of‐pocket payment	Descriptive statistics based upon a stratified sample	Weak	Moderate	2011‐2012	NS	Greece
Tountas et al^44^	Utilization by socioeconomic status	Multivariate logistic analysis	Weak	Moderate	2006	NS	Greece
**Quality of care**							
Berta et al^61^	Mortality rate	Multilevel	Strong	Weak	2009	NFP	Italy
Britto‐Arias et al^56^	Adherence guideline in colorectal cancer screening	Cohort study, relative frequencies with confidence intervals	Weak	Moderate	2007‐2013	NS	Austria
Gobillon and Milcent^57^	Mortality rate	Survival analysis: cox model	Strong	Moderate	1998‐2003	FP	France
Gusmano et al^58^	Rehospitalization rates	Step by step regression models	Moderate	Moderate	2010	NS	France
Louis et al^54^	Inappropriate medical admissions	Descriptive statistics	Weak	Weak	2001‐2005	NS	Italy
Moscone et al^60^	Readmission and death within 30 days	Multivariate OLS regression	Moderate	Weak	2005‐2007	NS	Italy
Owusu‐Frimponget al^63^	Patient satisfaction on accessibility	Mixed method: semistructured interviews + cross‐sectional survey using ANOVA	Weak	Weak	X	NS	United Kingdom
Pérotin et al^62^	Patients experience	Two‐stage switching regression model (incl. fixed effects)	Strong	Moderate	2007‐2008	ISTC	United Kingdom
Quercioli, Messina, Basu, McKee, Nante, and Stuckler^59^	Avoidable mortality	Region‐specific fixed effects	Strong	Strong	1993‐2003	NS	Italy
Stroffolini et al^55^	Compliance to the antenatal hepatitis B screening program	Multivariate logistic regression based upon a survey	Weak	Weak	2001	NS	Italy
**Multiple dimensions: accessibility, quality of care, and/or efficiency**							
Preti et al^48^	Characteristics of patients, patterns of care, and discharges	Chi‐square or Fischer exact test	Weak	Weak	Data collection 2001‐2005	Psych.	Italy
Berta et al^23^	Cream skimming^,^ readmission technical efficiency	SFA	Moderate	Strong	1998‐2007	FP + NFP	Italy
Fattore et al^34^	Regional physical mobility, LOS	Logistic regression + multilevel	Strong	Strong	2009	NS	Italy
Kondilis et al^36^	Bed capacity, occupancy rate, nursing staff rate, LOS, and payment per discharge	Confidence interval analysis	Weak	Moderate	2001‐2003	FP	Spain
Street et al^32^	Patients from deprived versus affluent regions, LOS	Within‐HRG differences with *t* test	Weak	Weak	2006/2007	ISTC	United Kingdom
Tiemann and Schreyogg^21^	Technical and controlled for mortality	Two‐stage DEA	Strong	Strong	2002‐2006	FP + NFP	Germany

Abbreviations: ANOVA, analysis of variance; COLS, corrected ordinary least squares; DEA, data envelopment analysis; Diff‐in‐Diff, difference‐in‐difference; FE, fixed effect; FP, for‐profit; HRG, Healthcare Resource Groups; ISTC, independent sector treatment centers; IVs, instrumental variables; LOS, length of stay; NFP, not‐for‐profit; NS, not specified; OLS, ordinary least squares; Psych., (private) psychiatric hospitals; SFA, stochastic frontier analysis.
